# Underground Architects of Resilience: Biodiversity and Role of Arbuscular Mycorrhizal Fungi in Date Palm (*Phoenix dactylifera* L.) Productivity and Adaptation to Arid Environments

**DOI:** 10.3390/jof12070502

**Published:** 2026-07-09

**Authors:** Elmostafa Gagou, Meryem Ben Moumen, Khadija Chakroune, Mondher El Jaziri, Abdelkader Hakkou

**Affiliations:** 1 Laboratory of Bioresources, Biotechnology, Ethnopharmacology and Health, Faculty of Sciences, Université Mohamed Premier, BV Mohammed VI BP 717, Oujda 60000, Morocco; meryem.benmoumen@ump.ac.ma (M.B.M.); khadija.chakroune@ump.ac.ma (K.C.); kadahakkou@yahoo.fr (A.H.); 2 Laboratory of Plant Biotechnology, Université Libre de Bruxelles, 6041 Gosselies, Belgium; mondher.el.jaziri@ulb.be

**Keywords:** arbuscular mycorrhizal fungi (AMF), arid environments, biofertilizer, *Phoenix dactylifera*, rhizosphere, stress tolerance, sustainable agriculture

## Abstract

The date palm is an icon of resilience in arid ecosystems, yet its survival is increasingly challenged by climate change and disease. Belowground, a powerful symbiotic solution exists: Arbuscular Mycorrhizal Fungi (AMF). This review synthesizes eight decades of research, from the pioneering observations in 1940 to contemporary studies, to build a comprehensive understanding of this critical partnership. We explore the vast, largely untapped biogeographic diversity of AMF, from the oases of North Africa to the deserts of Arabia, and detail the physiological and molecular mechanisms that underpin AMF-mediated tolerance to salinity, drought, and pathogens like *Fusarium oxysporum*. Evidence from individual studies indicates that AMF inoculation can increase plant biomass by more than 100% under specific stress conditions and substantially reduce Bayoud disease severity, although the magnitude of these effects varies with fungal identity, host cultivar, and experimental conditions. We then bridge this fundamental knowledge to cutting-edge biotechnological applications, including the design of precision inoculants and scalable in vitro production systems. We conclude by outlining a strategic vision for future research, focusing on the development of cultivar-specific consortia and the integration of multi-omics, to translate this fundamental ecological interaction into robust, sustainable agricultural solutions.

## 1. Introduction

The date palm (*Phoenix dactylifera* L.) constitutes a cornerstone of civilization and agriculture in the world’s most arid regions. Its socio-economic significance is immense, with global production reaching 9.2 million tonnes cultivated across approximately 1.27 million hectares in 2022 [1]. With an estimated 100 million trees worldwide and a staggering diversity of over 5000 cultivars, the date palm industry represents a global market valued at over $9 billion, providing a primary source of income for millions [2]. For many rural communities in North Africa and the Middle East, date cultivation is not merely a crop but a fundamental component of their cultural heritage and economic stability, a fact recognized by its inscription on the UNESCO list of Intangible Cultural Heritage [3].

The cultivation of date palm is intrinsically linked to a demanding climate. It thrives in regions characterized by long, intensely hot summers with low humidity and minimal rainfall during fruit ripening, and it can tolerate extreme temperatures exceeding 50 °C [4,5]. This unique adaptation allows it to flourish where few other crops can survive, making it a symbol of resilience.

Beyond its economic value, the date palm serves a critical ecological function as a keystone species in oasis agroecosystems. Its canopy creates a protective microclimate, a buffer against scorching sun and desiccating winds, that enables the establishment of a multi-layered agroforestry system. This “oasis effect” allows for the cultivation of other fruit trees, forages, and vegetables in its shade, thus fostering biodiversity and optimizing the use of scarce water resources [6]. Furthermore, the date palm plays an important role in combating desertification by stabilizing soil and preventing wind erosion [7].

Despite its remarkable adaptations, date palm cultivation is strongly constrained by water scarcity and soil salinization, two major abiotic stresses that severely limit its productivity [8,9,10,11]. Furthermore, date palms play a significant role in mitigating climate change, as they absorb carbon dioxide to a markedly greater extent than other tree species owing to their large biomass and long lifespan [12,13]. Estimates suggest that a mature date palm can sequester approximately 70–200 kg CO_2_ per year, depending on tree age, environmental conditions, and management practices. This sequestration rate significantly outperforms traditional temperate orchard crops such as apple and pear, which typically range from 10 to 25 kg CO_2_ per tree per year [14,15], and approaches the carbon-fixing capacity of certain fast-growing forestry species such as Eucalyptus, poplar, and willow during their early growth stages (10–40 kg CO_2_ per tree per year) [16,17]. This relatively high carbon sequestration potential is mainly attributed to the large biomass, perennial growth habit, and long lifespan of date palm trees, making them an important component of carbon storage in arid and semi-arid agroecosystems [13,18,19].

Concurrently, biotic threats, particularly the devastating Bayoud disease caused by *Fusarium oxysporum* Schltdl. f. sp. *albedinis* (Kill. & Maire) W.L. Gordon (Foa), have decimated millions of trees and threaten the genetic diversity of valuable cultivars, especially in Morocco and Algeria [20,21,22].

In the quest for sustainable solutions, the scientific community has turned its attention to the plant’s own microbiome. Among the myriad of microorganisms inhabiting the rhizosphere, AMF stand out as key symbiotic partners. These obligate biotrophs form a mutualistic association with the roots of over 80% of terrestrial plants, including the date palm [23,24,25]. The establishment of this symbiosis is a tightly coordinated process initiated by a molecular dialog between the two partners: root-derived strigolactones stimulate fungal spore germination and hyphal branching, while the fungus reciprocates with diffusible signals, including lipochitooligosaccharides that prime the root for symbiotic colonization. Once contact is established, the fungus extends its hyphal network into the soil, acting as an extension of the plant’s root system to enhance the uptake of water and poorly mobile nutrients, particularly phosphorus. In return, the plant allocates a substantial share of its photosynthetically fixed carbon to the fungus; while this exchange was long considered to occur solely as sugars, more recent evidence shows that the plant also transfers lipids that are essential to fungal development, since AMF lack the enzymatic machinery for de novo fatty acid synthesis and depend entirely on this host-derived supply [26,27,28].

This symbiotic relationship provides numerous benefits to host plants, including improved plant growth and nutrient acquisition, enhanced tolerance to abiotic stresses such as drought and salinity, and increased resistance to soil-borne pathogens through multiple physiological, biochemical, and molecular mechanisms [29,30,31].

Pioneering work in this area can be traced to Sabet (1940) [32], who published the first documented observation of a mycorrhizal habit in date palm. Working on cultivated date palms in Egypt, Sabet’s microscopic analysis revealed the presence of non-septate fungal hyphae within the root cortex, forming intercellular networks and highly branched intracellular arbuscules. He noted that these structures, the primary sites of nutrient exchange, invaginated the host cell’s plasma membrane without penetrating it, a key feature of this mutualism. By documenting these intricate biotrophic structures, Sabet provided the first definitive proof that this iconic crop actively engaged in an intimate symbiosis. Despite this early observation, it is primarily over the last two decades that research has intensified, exploring the diversity, function, and application of these beneficial fungi. Across major date-growing regions, the AMF communities associated with date palm rhizospheres exhibit pronounced biogeographic structuring. In North African oases, the genera *Glomus* Tul. & C. Tul., *Funneliformis* C. Walker & A. Schüßler, *Rhizophagus* P.A. Dang., *Acaulospora* Gerd. & Trappe, and *Scutellospora* C. Walker & F.E. Sanders are typically dominant, whereas the Arabian Peninsula harbors additional desert-adapted taxa such as *Diversispora*, *Paraglomus* J.B. Morton & Redecker, and *Ambispora*, including several site-specific lineages rarely reported elsewhere [33,34,35,36,37].

This review provides a comprehensive synthesis of the scientific knowledge accumulated over several decades on the AMF–date palm association. By analyzing the available peer-reviewed literature, we will cover the diversity and geographical distribution of AMF, their crucial role in mitigating abiotic and biotic stresses, and the biotechnological applications paving the way for a more sustainable and resilient date palm agriculture. As AMF rarely act in isolation within the rhizosphere, we also consider, where relevant, their interactions with other beneficial microorganisms including plant growth-promoting rhizobacteria, endophytic fungi, and organic amendments such as compost as complementary partners that can modulate AMF establishment and efficacy, rather than as independent subjects of this review.

Although the role of AMF in date palm cultivation has been previously reviewed, notably by Al-Karaki (2013) [38], earlier syntheses mainly focused on the agronomic benefits of AMF inoculation, including improved nutrient acquisition, plant growth, and sustainable management practices. Since then, substantial advances have been made in understanding the physiological, biochemical, and molecular mechanisms underlying AMF-mediated stress tolerance in date palm. In contrast to previous reviews, the present review provides an integrated and updated analysis of how AMF enhance date palm resilience to major abiotic and biotic constraints, including salinity, drought, and Bayoud disease, with particular emphasis on ion homeostasis, osmotic adjustment, antioxidant defense, and mycorrhiza-induced resistance.

## 2. Diversity and Distribution of AMF in the Date Palm Rhizosphere

The rhizosphere represents a hotspot of microbial diversity where complex interactions occur between plant roots and soil microorganisms. In the case of the date palm, a keystone species of arid and oasis ecosystems, this complex microbiome including AMF plays a crucial role in plant adaptation, nutrient acquisition, and survival under harsh desert conditions [39,40,41]. Over several decades, studies conducted across different date-growing regions have revealed a diversity of AMF species associated with date palm roots. However, their composition and abundance vary widely depending on geographic location, climate, soil characteristics, and agricultural management practices [23,42,43].

A comprehensive synthesis of AMF taxa documented in date palm rhizospheres across major date-growing regions worldwide is provided in Table 1**,** integrating data from studies conducted between 2006 and 2025. Despite their diversity, a limited number of genera consistently dominate AMF communities in date palm rhizospheres. Across major date-growing regions, the most frequently reported genera include *Glomus* (with approximately 15–20 morphologically described species reported across date-growing regions), *Funneliformis* (2–3 species, predominantly *F. mosseae* and *F. coronatus*), *Rhizophagus* (mainly *Rhizophagus irregularis* (Błaszk., Wubet, Renker & Buscot) C. Walker & A. Schüßler sensu lato), *Acaulospora* (4–6 species), *Gigaspora* (2–3 species) and *Scutellospora* (2–4 species) [33,44,45,46,47,48,49,50].

### 2.1. Geographic Distribution and Biogeographic Patterns

Although several AMF taxa are considered cosmopolitan, increasing evidence indicates that AMF communities exhibit strong biogeographic structuring driven by environmental filtering, soil characteristics, and host plant identity [43,57].

In North African oasis systems, members of the Glomeraceae family are typically dominant. For example, in the Ziz Valley of Morocco, Bouamri et al. (2006) [34] reported several AMF species associated with date palm rhizospheres, including *Glomus mosseae* T.H. Nicolson & Gerd. [currently *Funneliformis mosseae* (T.H. Nicolson & Gerd.) C. Walker & A. Schüßler], *G. fasciculatum* (Thaxt.) Gerd. & Trappe, *G. constrictum* Trappe, *G. aggregatum* N.C. Schenck & G.S. Sm., and *G. macrocarpum* Tul. & C. Tul., together with representatives of the genera *Acaulospora* Gerd. & Trappe and *Scutellospora* C. Walker & F.E. Sanders. Similar community structures, characterized by the dominance of Glomeraceae taxa, particularly *Glomus*, *Funneliformis*, and *Rhizophagus*, have been reported in other Moroccan oasis ecosystems including the Tafilalet–Zagora region [51,52] and the Drâa-Tafilalet oasis [47,48,49]. It should be noted, however, that *Acaulospora* Gerd. & Trappe (family Acaulosporaceae) and *Scutellospora* C. Walker & F.E. Sanders (family Gigasporaceae) are also consistently detected in date palm rhizospheres across these regions but belong to distinct fungal families within the phylum Glomeromycota, separate from the Glomeraceae *sensu stricto*. More recent work conducted in the Figuig oasis by Gagou et al. (2025) [44], combining morphological identification with molecular markers (LSU and ITS sequencing), revealed an even richer AMF community dominated by genera of the Glomeraceae (*Glomus*, *Funneliformis*, and *Rhizophagus*) as well as members of the Acaulosporaceae.

In Saharan environments of Algeria, particularly in the Ouargla region, surveys have revealed diverse AMF communities associated with date palm rhizospheres, including *Glomus*, *Scutellospora*, *Acaulospora*, *Rhizophagus*, *Gigaspora*, *Funneliformis*, *Claroideoglomus*, and *Racocetra*, with *Glomus* and *Scutellospora* reported as dominant genera [55]. These assemblages appear to be shaped by strong ecological filtering associated with extreme aridity, high soil alkalinity, and nutrient limitation, resulting in communities with relatively low species richness dominated by stress-tolerant Glomeraceae taxa exhibiting high sporulation capacity. Comparable patterns of stress-driven community simplification and Glomeraceae dominance have also been documented in Tunisian oasis ecosystems. In the Saharan oases of Tozeur, Chebaane et al. (2020) [49] reported AMF communities associated with date palm roots characterized by low generic diversity, including *Funneliformis coronatus* (Giovann.) C. Walker & A. Schüßler, *R. irregularis*, *Dominikia disticha* (T.H. Nicolson & Schenck) Oehl, Błaszk. & Sieverd, and *Albahypha drummondii* (Błaszk. & Renker) Sieverd., Oehl, B.T. Goto & G.A. Silva. These fungal assemblages were associated with high levels of root colonization exceeding 70%, indicating that symbiotic efficiency can be maintained despite low AMF species richness under saline and arid conditions [49].

Despite this historical precedent, Sabet’s (1940) [32] foundational observation having itself been made on Egyptian date palms, no dedicated molecular or morphological survey of AMF communities in Egyptian date palm rhizospheres appears to have been published over the past two decades, representing a notable gap given the country’s major date-growing status.

Studies on AMF associated with date palm in the Arabian Peninsula and Persian Gulf region remain remarkably scarce and geographically uneven despite the major agro-economic importance of date palm cultivation in this part of the world. The available literature is largely concentrated in Oman and, to a lesser extent, Saudi Arabia, whereas investigations in the United Arab Emirates, Kuwait, Bahrain, Yemen, and Iraq remain very limited or absent regarding date palm–AMF interactions [58]. In Oman, Al-Yahya’ei et al. (2011) [35] documented unique AMF assemblages associated with date palms, characterized by a high degree of molecular diversity and several site-specific taxa adapted to hyper-arid desert environments. Subsequent work by Symanczik et al. (2014) [33], using single-spore cultures and molecular phylogenetic analyses (LSU rDNA), identified several desert-adapted species including *Claroideoglomus drummondii* (Błaszk. & Tadych) C. Walker & A. Schüßler, *Diversispora aurantia* Błaszk., Ryszka, Kovács & Buscot, *Diversispora spurca* (C.M. Pfeiff., C. Walker & Bloss) C. Walker & A. Schüßler, and *Funneliformis africanum* Oehl, Tchabi, Hountondji, Lawouin, Coyne & Sieverd. These findings highlight the presence of specialized AMF lineages adapted to the nutrient-poor sandy soils of the Arabian Peninsula desert ecosystems.

Early observations in the Qassim oases of Saudi Arabia by Khudairi (1969) [59] first reported AMF colonization in date palm groves. More recent surveys conducted at Al-Jamil Farm revealed mycorrhizal colonization rates ranging from 28% to 72% in plant species associated with date palm ecosystems, together with spore densities of 95–130 spores per 100 g of dry soil [56]. In Qatar, the first dedicated survey of AMF communities in arid habitats was published in 2022 and identified *C. drummondii* and *R. irregularis* as the dominant taxa [36]. Consequently, the apparently low AMF diversity reported in earlier syntheses [38] should be interpreted cautiously, as it likely reflects insufficient sampling effort rather than genuinely low fungal diversity. Overall, the Persian Gulf region remains one of the least explored areas worldwide with respect to AMF–date palm interactions.

Overall, the available studies indicate that AMF communities associated with date palms are not randomly distributed but instead reflect complex interactions among environmental conditions, soil physicochemical properties, host plant characteristics, and evolutionary history. However, the biogeographic patterns currently emerging from the literature remain strongly influenced by the unequal distribution of research efforts across regions, with the Persian Gulf and the broader Arabian Peninsula representing major priorities for future AMF biodiversity surveys.

### 2.2. Factors Influencing AMF Community Structure

The structure of AMF communities in date palm rhizospheres is shaped by multiple interacting factors, including environmental and edaphic conditions, agricultural practices, host plant genetics, and seasonal dynamics.

#### 2.2.1. Environmental and Edaphic Factors

Soil pH strongly influences nutrient availability and microbial functioning, primarily by governing the solubility of phosphorus and micronutrients such as zinc, iron, and manganese, which become progressively less available to plant roots as pH rises above neutral [60,61]. Date palm plantations are often located in alkaline soils (pH > 8), a condition that constrains root-mediated nutrient acquisition and can therefore reinforce the plant’s dependency on the mycorrhizal pathway for phosphorus uptake. Under such conditions, AMF communities tend to be dominated by a restricted set of pH-tolerant, stress-adapted taxa within the Glomeraceae, a pattern of community simplification comparable to that observed under high salinity (see below) [53].

Soil salinity is another critical driver that significantly affects AMF dynamics. High salt concentrations generally inhibit root colonization rates while selecting for salt-tolerant taxa [62,63]. Experimental evidence suggests that root colonization in date palms can decrease by 30–50% under severe salinity (200–250 mM NaCl). Nevertheless, specific halotolerant species, such as *F. mosseae*, *F. coronatus*, and *R. irregularis*, often exhibit high resilience and maintain symbiotic efficiency (colonization rates above 70%) under these stressful conditions [64,65]. These findings are corroborated by field studies in Tunisian and Omani oases, where a strong negative correlation was observed between soil electrical conductivity (EC), the standard proxy for soil salinity, and both spore density and infective propagules; in Tunisian oases where EC reached 7.6 dS m^−1^, root colonization declined markedly relative to less saline sites, mirroring the 30–50% reduction reported above under controlled NaCl exposure [35,49]. Recent metagenomic analyses confirm that salinity levels, alongside plant genotype, represent the primary drivers of fungal community shifts in the date palm rhizosphere [66].

Nutrient availability, particularly phosphorus (P), strongly regulates the establishment and maintenance of the AMF symbiosis. Under high soil P concentrations, host plants downregulate their dependence on AMF to conserve carbon, thereby actively suppressing root colonization. This plant-mediated feedback is driven by physiological shifts, notably the reduced root exudation of signaling molecules such as strigolactones, which limits fungal proliferation [25,67]. In date palm agroecosystems, field observations confirm this dynamic; soil P content has been shown to be significantly negatively correlated with both AMF spore density and root colonization intensity in oasis environments [45,53].

#### 2.2.2. Agricultural Practices

Agricultural management strongly modulates AMF diversity and functioning in date palm agroecosystems. Beyond the intrinsic salinity of arid soils, the irrigation regime, particularly the common practice of using saline groundwater in arid oasis systems, can exacerbate salt accumulation and further suppress non-adapted AMF populations.

Soil disturbance caused by intensive management is a second critical factor. Conventional tillage physically fragments the extraradical mycelium network and disrupts the continuity between colonized root fragments and spores [68,69]. This mechanical disruption reduces hyphal length density by 40–60% and root colonization by 15–30% compared with no-till systems [70,71]. Chemical inputs further compound these negative effects; the application of broad-spectrum fungicides can cause reductions of up to 50% in AMF colonization, with recovery times exceeding one full growing season [72]. Similarly, excessive chemical phosphorus fertilization triggers the aforementioned host-mediated downregulation of the symbiosis, demonstrating that intensive mineral inputs directly antagonize indigenous AMF populations [53].

Conversely, sustainable practices such as organic amendments (particularly composts) improve soil structure and stimulate indigenous microbial communities, thereby supporting AMF proliferation. The synergistic effect of AMF combined with compost is detailed in Section 3.1.3. Furthermore, traditional oasis agroforestry systems, with their multi-layered vegetation, support higher AMF generic diversity than monoculture plantations [58], reflecting diverse root exudate profiles and more stable soil microenvironments.

#### 2.2.3. Host Plant Genetics and Cultivar Specificity

Host plant genotype represents another major driver of AMF community composition. Different date palm cultivars produce distinct root exudate profiles containing sugars, amino acids, and secondary metabolites that influence AMF recruitment [73,74]. Among these signaling molecules, strigolactones play a central role by stimulating AMF spore germination and hyphal branching at very low concentrations [75]. Flavonoids such as quercetin and luteolin can also act as chemoattractants for AMF, whereas certain phenolic compounds may inhibit colonization, thereby creating selective pressures that favor compatible fungal partners [76,77].

Studies conducted in Southern Arabia have demonstrated that different date palm cultivars host distinct AMF assemblages, with species such as *G. sinuosum* and *Claroideoglomus etunicatum* (W.N. Becker & Gerd.) C. Walker & A. Schüßler frequently dominating local communities [35]. Beyond community composition, the host genotype also governs the functional outcomes of the symbiosis. Research in Moroccan oases highlights that mycorrhizal status, root colonization intensity, and subsequent symbiotic effectiveness (e.g., nutrient uptake efficiency and biomass accumulation) vary significantly across traditional cultivars such as ‘Boufeggous’ and ‘Majhool’ [34,46]. Some cultivars exhibit higher mycorrhizal dependency and responsiveness than others under environmental stress. This high intra-specific variability underscores that a “one-size-fits-all” approach is inadequate for biofertilization; instead, it reinforces the need for cultivar-specific inoculation strategies, pairing targeted date palm varieties with compatible, highly efficient indigenous AMF strains to maximize agricultural resilience in oasis ecosystems.

### 2.3. Seasonal Dynamics

The AMF–date palm symbiosis is highly dynamic and influenced by seasonal changes in climate, soil moisture, and plant physiology [78,79]. Temperature and soil water availability are major drivers of seasonal AMF colonization dynamics [80]. Bouamri et al. (2014) [51] documented pronounced seasonal variation in the date palm groves of Tafilalet, Morocco, a region characterized by extreme thermal amplitudes ranging from −1.5 °C in January to 50 °C in July. Root colonization rates varied from 7% to 60% depending on the site and season, with maximum colonization observed during the wetter periods (April and October), which coincided with active vegetative growth and favorable soil moisture conditions. In contrast, minimum colonization levels occurred during the dry summer period when soil water availability declined sharply. Spore density exhibited an inverse seasonal pattern, ranging from 331 to 1431 spores per 100 g of soil and increasing by 36–47% during the dry season (spring and summer) relative to wetter periods, suggesting a functional shift from active root colonization to reproductive sporulation under environmental stress. Species richness also showed marked seasonal variation: 12 of the 15 identified morphotypes sporulated in April, 10 in July and October, but only six in January, indicating that winter low temperatures suppress both colonization and sporulation activity. Notably, species belonging to the genus *Glomus* sporulated throughout the year, whereas *Acaulospora* and *Scutellospora* species were mainly detected during seasonal transition periods (spring and autumn), reflecting genus-specific phenological strategies. This trade-off between active colonization and sporulation, together with contrasting seasonal phenologies among AMF taxa, appears to represent an important adaptive mechanism enabling AMF persistence in arid oasis ecosystems.

Similarly, in the arid ecosystems of the Arabian Peninsula, AMF spore density tends to increase during the extreme heat and aridity of summer, reflecting a specialized survival strategy under unfavorable conditions [35,81]. During these stress periods, attenuated carbon allocation from the host plant often triggers a phenological shift in the fungus from vegetative hyphal expansion to reproductive spore production. These thick-walled spores persist in the soil matrix until the return of favorable environmental conditions, enabling the rapid recolonization of roots in subsequent growing seasons [82,83]. These seasonal patterns have significant practical implications for AMF inoculation strategies. Inoculation during periods of active root development, particularly in spring, generally yields higher colonization rates and faster establishment of functional symbioses, whereas winter applications are typically less effective due to reduced metabolic activity [84,85]. Understanding these temporal dynamics is therefore essential for maximizing the agronomic benefits of AMF in date palm cultivation.

## 3. Role of AMF in Abiotic Stress Tolerance

**Figure 1:**
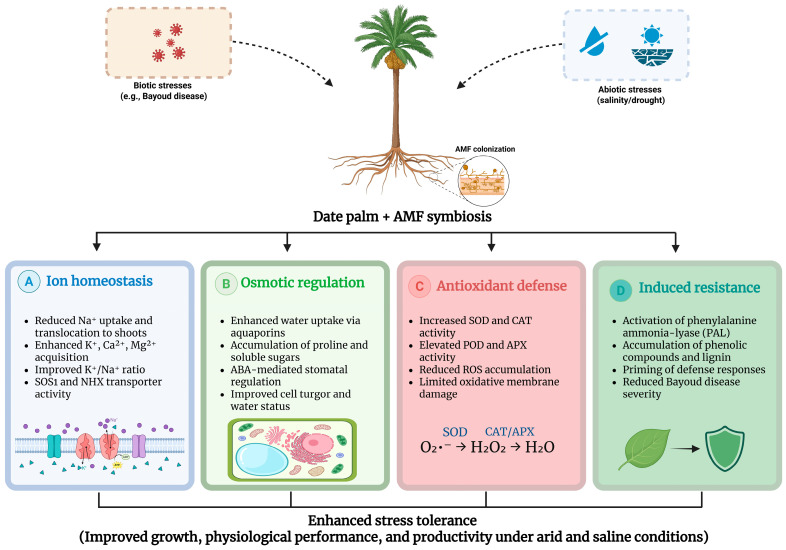
Conceptual framework illustrating the major mechanisms involved in AMF-mediated stress tolerance in date palm. AMF colonization enhances date palm resilience to salinity, drought, and Bayoud disease through four interconnected mechanisms. (**A**) Ion homeostasis is improved by reducing Na^+^ accumulation, enhancing K^+^, Ca^2+^ and Mg^2+^ uptake, maintaining a favorable K^+^/Na^+^ ratio, and regulating ion transport systems such as SOS1 and NHX. (**B**) Osmoregulation and water relations are strengthened through enhanced water uptake, aquaporin activity, ABA-mediated signaling, and accumulation of compatible solutes including proline and soluble sugars. (**C**) Antioxidant defense is reinforced by increasing the activity of reactive oxygen species (ROS)-scavenging enzymes, including superoxide dismutase (SOD), catalase (CAT), peroxidases (POD), and ascorbate peroxidase (APX), thereby limiting oxidative damage. (**D**) Mycorrhiza-induced resistance promotes activation of defense-related pathways, including phenylalanine ammonia-lyase (PAL), accumulation of phenolic compounds, lignification, and suppression of pathogen development. Together, these mechanisms improve plant growth, physiological performance, and stress resilience under arid and saline environments.

Figure 1 provides a conceptual overview of the four principal mechanisms through which AMF confer stress tolerance in date palm, each of which is examined in detail in the sections that follow.

Oasis ecosystems are characterized by harsh environmental conditions, notably high salinity and prolonged drought. AMF enhance date palm resilience under these conditions primarily by improving nutrient acquisition, regulating ionic balance, and reinforcing antioxidant defenses.

### 3.1. Salinity Tolerance

Soil salinization is a major and growing threat to date palm productivity worldwide. AMF inoculation consistently improves plant performance under salt stress, a phenomenon documented across numerous species [63,86,87]. In date palms specifically, experimental evidence confirms that AMF enhance growth, nutrient uptake, and physiological resilience under saline conditions, with protective effects that vary in magnitude depending on fungal identity, cultivar genotype, and the presence of complementary amendments [49,88,89,90].

#### 3.1.1. Improved Ion Homeostasis

AMF-inoculated date palms selectively reduce the uptake and translocation of toxic ions such as Na^+^ and Cl^−^ to aerial tissues while enhancing the acquisition of essential mineral nutrients, including K^+^, Ca^2+^, and Mg^2+^ [64,89,91]. In the cultivar ‘Medjool’ exposed to 240 mM NaCl, mycorrhizal seedlings showed significantly lower Na^+^ accumulation and higher K^+^ and Ca^2+^ concentrations compared with non-mycorrhizal plants, resulting in a markedly improved K^+^/Na^+^ ratio [65]. Similar ionic regulation was independently confirmed in ‘Boufeggouss’ seedlings under identical salinity conditions [89], indicating that this response, while cultivar-dependent in magnitude, represents a consistent feature of AMF-mediated salt tolerance in date palm.

At the molecular level, AMF coordinate ion transport through the simultaneous upregulation of multiple transporter families: plasma membrane Na^+^/H^+^ antiporters (SOS1), which extrude Na^+^ from the cytosol and restrict xylem loading; vacuolar Na^+^/H^+^ exchangers (NHX), which sequester Na^+^ in vacuoles; and HKT1 transporters, which unload Na^+^ from the xylem stream in shoots [92,93]. The expression patterns of these transporters vary by plant species and tissue type, explaining the quantitatively different ionic responses observed across date palm cultivars subjected to the same AMF treatment. In addition, AMF upregulate aquaporin genes (*PIP*) and H^+^-ATPase genes (*VHA-B*), facilitating osmotic adjustment at the cellular level [92,94].

#### 3.1.2. Integrated Physiological and Biochemical Responses to Salinity Stress

The beneficial effects of AMF on date palm salinity tolerance result from a complex interplay of physiological and biochemical adjustments. Rather than acting through a single pathway, AMF coordinate responses related to growth maintenance, osmotic balance, and oxidative stress mitigation, thereby enhancing plant performance under saline conditions. The principal mechanisms involved are outlined below.

Growth and photosynthesis: Mycorrhizal date palm seedlings exposed to severe salt stress (up to 240 mM NaCl) consistently display greater shoot and root biomass, higher leaf area, and improved stomatal conductance and chlorophyll content compared with non-mycorrhizal controls [64,65,89]. A large-scale meta-analysis covering hundreds of AMF–plant studies confirmed that AMF-mediated biomass gains become more pronounced as salinity intensity increases, and that the magnitude of these effects is significantly influenced by both fungal identity and host plant traits [95]. In date palm specifically, locally adapted AMF isolates consistently outperform exotic strains in sustaining photosynthetic performance under stress [96], underscoring the critical importance of biogeographic origin in inoculant selection.

Osmotic adjustment: AMF promote the accumulation of compatible solutes, notably proline and soluble sugars, under saline conditions [89,97]. However, the relationship between stress intensity and osmolyte accumulation is non-linear and functionally informative. Meta-analytical evidence indicates that proline and catalase (CAT) accumulation occur predominantly under moderate salinity stress, whereas superoxide dismutase (SOD) and peroxidase (POD) are significantly elevated regardless of stress intensity [95]. Consequently, under severe stress with highly effective AMF colonization, proline levels may paradoxically stabilize rather than continue to rise, an observation reported by Ait-El-Mokhtar et al. (2020) [65] that reflects successful alleviation of osmotic stress rather than diminished defense capacity. This distinction is critical for correctly interpreting osmolyte data as biomarkers of symbiotic efficiency yet has been largely overlooked in prior date palm reviews.

#### 3.1.3. Synergistic Effects with Organic Amendments

In date palm, the dual application of a native AMF consortium with green waste compost consistently outperforms either treatment alone across multiple independent studies [47,65,90]. In the cultivar ‘Boufeggouss’ exposed to 240 mM NaCl, this dual treatment improved all measured parameters, namely shoot and root biomass, stomatal conductance, leaf water potential, mineral nutrition (P, K, N, Ca), osmolyte accumulation, and antioxidant defenses, more effectively than either treatment in isolation [65]. Importantly, a longitudinal study by Ait-El-Mokhtar et al. (2022) [90] extending observations over 10 to 14 months confirmed that these benefits are sustained over time, with the greatest improvement in chlorophyll b content (+56%) recorded under the dual treatment, adding a temporal dimension that single time-point studies cannot capture.

Beyond date palm, the generality of this synergistic mechanism is supported by independent evidence from other crops. Lahbouki et al. (2023) [98] demonstrated in maize under salinity that AMF combined with organic amendments produced superior K^+^/Na^+^ ratios and antioxidant responses compared with single treatments, while Hasanuzzaman et al. (2022) [99] confirmed in a comprehensive cross-species review that compost improves ionic homeostasis, photosynthetic apparatus, and antioxidant capacity under salinity across multiple agricultural species, providing broad ecological validity to the date palm-specific findings.

This synergy reflects mechanistically complementary actions operating at different scales. At the plant level, AMF enhance phosphorus and water acquisition through their extensive extraradical hyphal networks. At the soil level, compost improves soil structure, increases cation exchange capacity, stimulates indigenous microbial activity, and enhances nutrient availability conditions that in turn support AMF establishment and colonization efficiency [65,89,99]. An important and often overlooked finding is that compost application can slightly reduce AMF root colonization rates, yet the combined AMF + compost treatment still outperforms AMF alone across all physiological parameters [65,90], suggesting that the soil-level benefits of compost more than compensate for any reduction in colonization intensity. The valorization of agricultural by-products including date palm residues as locally produced compost paired with indigenous AMF therefore represents a biologically integrated, low-cost, and ecologically coherent strategy for managing salt-affected soils in arid agroecosystems, fully aligned with the principles of circular agriculture and sustainable intensification.

### 3.2. Drought Tolerance

Water scarcity is a defining feature of the arid ecosystems where date palms grow, and AMF symbiosis significantly enhances date palm drought tolerance through three integrated mechanisms: improved water relations mediated by aquaporins and root system remodeling, enhanced nutrient acquisition via the extraradical hyphal network, and strengthened antioxidant defenses coordinated by ABA signaling [46,47,88,97,100]. The magnitude of these benefits depends on fungal identity, host cultivar, and the duration and intensity of water stress.

Under short-term drought, AMF protection operates primarily through improved plant water relations, detailed in Section 3.2.1 at the molecular level; this enhanced water homeostasis is supported by AMF-mediated regulation of host aquaporins (AQPs). Inoculation with *Rhizophagus intraradices* (N.C. Schenck & G.S. Sm.) C. Walker & A. Schüßler upregulates the fungal aquaporins GintAQPF1 and GintAQPF2 in extraradical mycelia and modulates host PIP2 phosphorylation status, thereby enhancing water channel activity under drought [101,102]. AMF symbiosis also elevates endogenous ABA, the “abiotic stress hormone”, which orchestrates stomatal closure, hydraulic conductivity adjustment, and aquaporin gene expression, a coordinated response particularly effective under severe drought [102,103]. Under long-term drought, antioxidant defense activation plays a major role, as detailed in Section 3.2.2. Briefly, *R. intraradices* outperformed *F. mosseae* in alleviating oxidative damage in date palm, with significantly lower H_2_O_2_ and MDA accumulation and higher SOD, CAT, APX, and G-POD activities in inoculated seedlings [100]. A recent meta-analysis confirmed across multiple plant species that AMF inoculation under drought significantly reduces H_2_O_2_, MDA, and electrolyte leakage, while modulating non-enzymatic antioxidants in a stress intensity-dependent manner [104].

Combining AMF with complementary biofertilizers further amplifies drought tolerance through multi-microbial synergy. AMF extend root water absorption through hyphae, PGPR produce ACC deaminase that reduces drought-induced ethylene stress, and compost enhances soil water retention and microbial activity [103].

#### 3.2.1. Improved Water Relations and Growth

Mycorrhizal symbiosis significantly enhances drought tolerance in date palm by improving plant water relations and sustaining growth under water-limited conditions. Meddich et al. (2015) [46] evaluated date palm seedlings cv. ‘Bouffgouss’ grown under two irrigation regimes corresponding to 75% and 25% of field capacity and compared non-mycorrhizal plants with plants inoculated with a native Aoufous AMF consortium or selected *Glomus* species. Under severe water deficit (25% field capacity), plants inoculated with the Aoufous consortium or *Glomus monosporus* produced shoot dry biomass about 2.4-fold higher than non-mycorrhizal controls, whereas *G. clarum* showed intermediate effects and *G. deserticola* was clearly less effective. Mycorrhizal plants also maintained a better water status, as reflected by higher relative water content and less negative leaf water potential than uninoculated plants. In particular, leaf water potential reached about −25.46 MPa in plants inoculated with *G. monosporus* and −28.61 MPa in those inoculated with the Aoufous consortium, compared with −35.03 MPa in non-mycorrhizal controls under severe drought. Likewise, stomatal resistance remained markedly lower in the most efficient mycorrhizal treatments (2.13–2.15 s cm^−1^) than in non-mycorrhizal plants (3.21 s cm^−1^), indicating improved stomatal regulation and maintenance of gas exchange under drought. These findings highlight that AMF-mediated drought tolerance in date palm is strongly dependent on fungal identity, with native inocula and selected efficient strains providing the greatest benefits for growth and water balance under deficit conditions.

#### 3.2.2. Biochemical Responses and Fungal Specificity

The plant’s biochemical responses to drought are also profoundly modulated by the AMF symbiosis [105,106,107,108]. Meddich et al. (2015) [46] observed that while the levels of total phenols and the activities of defense-related enzymes like peroxidase and polyphenol oxidase increased in the roots of all plants under severe water stress, these levels were consistently higher in mycorrhizal palms. This suggests an AMF-primed enhancement of the plant’s defense pathways.

Furthermore, the effectiveness of this symbiosis is strongly fungus-dependent. Under long-term drought, Benhiba et al. (2015) [100] showed that *R. intraradices* was more effective than *F. mosseae* in improving growth, limiting oxidative damage, and enhancing antioxidant enzymes in date palm seedlings. Similar strain-dependent differences are also observed between native AMF consortia and commercial or exotic strains inoculated on date palm [48,64,96,97,109]. Collectively, this evidence underscores that the careful selection of the most effective and locally adapted AMF strains is critical for maximizing drought tolerance benefits in date palm cultivation.

## 4. Role of AMF in Biotic Stress Tolerance: The Case of Bayoud Disease

### 4.1. AMF-Mediated Direct Defense Against Bayoud Disease

Bayoud disease, caused by Foa, remains the most devastating soil-borne disease of date palm, with massive losses in North African groves where chemical control has proven largely ineffective [20,110,111,112]. AMF have therefore emerged as promising biocontrol agents through pre-inoculation of seedlings, with the first experimental evidence provided by Jaiti et al. (2007) [50] and Abohatem et al. (2011) [113] in the susceptible cultivar ‘Boufeggous’, who demonstrated significant reductions in disease severity and improvements in shoot height and root biomass following AMF colonization.

More recent studies have confirmed the agronomic relevance of this symbiosis under conditions closer to field production. Gagou et al. (2025) [45] evaluated the effect of a locally adapted AMF consortium from the Figuig oasis (Morocco) on the highly susceptible cultivar ‘Boufeggous Gharas’ under controlled greenhouse conditions. Inoculated seedlings showed a near-complete suppression of disease symptoms and significant improvements in plant growth, including a 51% increase in shoot height and a doubling of total biomass. Root necrosis was also reduced by approximately 75% relative to infected non-mycorrhizal controls, demonstrating the strong protective capacity of AMF under these controlled conditions. As with most of the experimental evidence reviewed here (Table 2), this result has not yet been independently replicated or validated under field conditions, and its consistency across environments, AMF isolates, and cultivars remains to be established.

Subsequent studies provided mechanistic insight into this protection in date palm. Mycorrhizal colonization and Foa infection strongly activate the phenylpropanoid pathway, as shown by increased PAL activity, higher polyphenol and lignin contents in roots, and elevated POX activity in mycorrhizal seedlings compared to non-mycorrhizal controls [47,113,114]. These biochemical changes, together with the enhanced accumulation of hydroxycinnamic acid derivatives, were closely associated with lower disease incidence and reduced pathogen colonization, supporting the concept of mycorrhiza-induced resistance in date palm [45,113,114,115]. The control of Bayoud disease by AMF results from the integration of multiple complementary mechanisms. First, mycorrhizal symbiosis enhances nutrient acquisition and overall plant vigor, thereby strengthening the plant’s basal capacity to withstand pathogen attack. For example, Abohatem et al. (2011) [113] reported that mycorrhizal ‘Boufeggous’ seedlings displayed approximately a twofold increase in leaf P concentration and improved K^+^ uptake. Improved nutritional status is widely recognized as a critical factor enhancing plant resilience to biotic stress.

In parallel, AMF establish an extensive extraradical hyphal network in the rhizosphere, which contributes to both physical and ecological barriers against pathogen establishment. As reported by Jaiti et al. (2007) [50], AMF compete with Foa for root colonization sites and plant-derived carbon resources. This competition restricts pathogen germination and proliferation in the rhizosphere, thereby complementing the plant’s induced defense responses.

### 4.2. AMF in the Context of Other Beneficial Microorganisms: PGPR and Endophytes

Although this review primarily focuses on the role of arbuscular mycorrhizal fungi in date palm stress tolerance and health, AMF do not function in isolation within the rhizosphere. In natural soils, AMF rarely act in isolation but function within complex microbial networks whose combined activities shape plant performance and stress resilience [116,117,118,119]. For Bayoud disease, Ziane et al. (2023) [114] showed that combined inoculation of an AMF consortium with a PGPR consortium provided markedly better protection of date palm seedlings against Foa than either inoculant alone. Co-inoculation increased mycorrhizal colonization intensity about threefold and strongly activated PAL activity and increased polyphenol and lignin contents in roots [114]. These changes coincided with a pronounced reduction in disease incidence, severity, and mortality, highlighting how microbial consortia can amplify plant immune responses through coordinated biochemical and structural defenses [114].

Under abiotic stress, combining AMF with compost or PGPR also outperforms single applications, as detailed in Section 3.1.3 [65,89,90]. Anli et al. (2020) [97] established that the triple combination of AMF + PGPR + compost provided the greatest protection under drought, confirming the treatment hierarchy of AMF alone < AMF + compost < AMF + PGPR + compost across both salinity and drought conditions.

From a broader perspective, these findings support the concept of microbiome-assisted crop resilience, in which AMF act not as a single biofertilizer but as keystone organisms that connect plant, soil, and microbial functioning. Their extraradical hyphae create the mycorrhizosphere, a specific microhabitat that selectively recruits hyphae-associated bacteria and concentrates beneficial microbial activity around plant roots [116]. AMF also produce glomalin-related soil proteins, which improve soil aggregation, water retention, and microbial habitat quality properties particularly valuable in degraded oasis soils where soil structure represents a major limitation to date palm productivity. AMF-based microbial consortia therefore represent a promising strategy for sustainable date palm cultivation, offering an integrated biological response to the major constraints of Bayoud disease, salinity, and drought in arid environments.

Together, the experimental evidence reviewed in Section 3 and Section 4 demonstrates that AMF inoculation produces robust and quantifiable benefits across the major stresses affecting date palm. Table 2 synthesizes these quantitative effects on growth, physiological, biochemical, and stress-related responses, illustrating the magnitude and consistency of the symbiotic benefits across cultivars, AMF inocula, and stress types.

**Table 2:** Quantitative effects of AMF inoculation on growth, physiological, biochemical, and stress-related responses of date palm (*P. dactylifera*) under abiotic (salinity, drought) and biotic (Bayoud disease) stresses. Studies are organized by stress type. Detailed inoculum compositions, co-applied treatments, and experimental conditions are reported as in the original publications.

Stress Type	AMF Inoculum (Composition)	Co-Applied Treatment	Date Palm Cultivar	Quantitative Effects (vs. Non-Mycorrhizal Controls)	Experimental Conditions	Reference
Abiotic stress						
Salinity	Native AMF consortium isolated from Marrakech region, Morocco (*Glomus*, *Funneliformis*, *Rhizophagus*, *Acaulospora* spp.)	Green waste compost (locally produced)	Seedlings cv. ‘Boufeggous’ (9 months old)	Combined AMF + compost vs. control: +226% shoot dry biomass Reduced Na^+^ and Cl^−^ accumulation Reduced MDA and H_2_O_2_ +74% CAT activity Improved K^+^/Na^+^ ratio Improved leaf water potential and stomatal conductance	Greenhouse experiment 240 mM NaCl Duration: 5 months	Ait-El-Mokhtar et al. (2020) [65]
Salinity	Two native AMF complexes (from Zagora and Marrakech, Morocco) and two non-native monospecific isolates (*Rhizophagus irregularis*, *Funneliformis mosseae*)	None	Date palm seedlings (cv. not specified)	AMF-inoculated vs. control: Colonization rates: − 25–50% (0 g/L NaCl) − 16–34% (10 g/L NaCl) − 18–41% (20 g/L NaCl) +23.5% protein content Root biomass: +60% (0 g/L), +45% (10 g/L), +39% (20 g/L) Native AMF complexes generally more effective under salinity	Greenhouse experiment 0, 10, and 20 g/L NaCl Duration: 4 months	Outamamat et al. (2021) [96]
Drought	Aoufous native AMF consortium (from Aoufous palm grove, Morocco) *G. monosporus,* *G. clarum* *G. deserticola*	None	Seedlings cv. ‘Boufeggous’ (grown from germinated seeds)	Aoufous consortium and *G. monosporus* most effective: +140% (×2.4) shoot dry biomass Leaf water potential: − AMF: −25.5 to −28.6 MPa − Control: −35.0 MPa Stomatal resistance: − AMF: 2.13–2.15 s cm^−1^ − Control: 3.21 s cm^−1^ Mycorrhizal frequency > 60%	Greenhouse experiment 25% field capacity (severe drought) vs. 75% FC (well-watered) Duration: 36 weeks	Meddich et al. (2015) [46]
Drought	*R. intraradices* (single species) *F. mosseae* (single species)	None	Seedlings (cultivar not specified)	*R. intraradices* most effective under long-term drought: Reduced H_2_O_2_ and MDA accumulation Increased SOD, CAT, APX, G-POD activities Increased proline, soluble sugars, and protein contents Improved drought avoidance via enhanced antioxidant defense	Greenhouse experiment 25% field capacity (long-term drought stress) Duration: Long-term water deficit	Benhiba et al. (2015) [100]
Drought	Native AMF consortium (autochthonous from Moroccan oases; Species not specified)	Native PGPR consortium Local compost	In vitro plantlets cv. ‘Boufeggous’	Triple combination AMF + PGPR + compost most effective: +76% plant biomass +293% phosphorus uptake +208% soluble sugars +84% protein content Reduced H_2_O_2_ and MDA levels Improved soil organic matter and glomalin content	Field experiment under semi-arid climate Well-watered (75% FC) vs. drought (25% FC) Duration: Not specified	Akensous et al. (2022) [120]
Drought	Native AMF consortium (species not specified)	Native PGPR consortium	Seedlings cv. ‘Najdi’ (not specified Saudi Arabian cultivar)	AMF + PGPR most effective: +163% proline content under severe drought Improved relative water content (RWC) Reduced membrane permeability Enhanced antioxidant enzyme activities (POD, CAT, SOD, GST)	Greenhouse experiment 16 h light/8 h dark photoperiod 25–30 °C, 60–70% RH Duration: 8 months	Harkousse et al. (2021) [121]
Biotic stress—Bayoud disease (*Fusarium oxysporum* f. sp. *Albedinis,* Foa)						
Biotic (Foa)	Indigenous AMF consortium from Figuig oasis, Morocco (*Funneliformis*, *Glomus*, *Rhizophagus*, *Scutellospora*, *Acaulospora* spp.)	None	10-week-old seedlings cv. ‘Boufeggous Gharas’ (highly susceptible to Foa)	AMF + *Foa* vs. *Foa*-only control: +51% shoot height +61% root length +100% (×2) total biomass	Greenhouse-controlled conditions 25 °C, 70% RH, 16 h light/8 h dark Pre-inoculation with AMF before *Foa* spore challenge	Gagou et al. (2025) [45]
Biotic (Foa)	Four AMF treatments: Aoufous native consortium (from Aoufous palm grove, Morocco) *G. monosporus* *G. deserticola* *G. clarum*	None	Date palm seedlings (Aoufous consortium most effective; cultivar from Aoufous palm grove)	AMF + *Foa* vs. *Foa*-only control: 8–77% reduction in disease severity (depending on AMF isolate) Aoufous consortium most effective Increased peroxidase (POD) and polyphenol oxidase (PPO) activities Stimulated shoot height, biomass, and leaf number	Controlled greenhouse conditions *Foa* inoculation after AMF colonization Duration: Long-term post-inoculation	Jaiti et al. (2008) [115]
Biotic (Foa)	AMF consortium (31 species; isolated from wild plant rhizospheres, Morocco): 16 species of *Rhizophagus* 10 species of *Acaulospora* 2 species of Entrophospora (*Diversispora*) 3 species of other genera	PGPR consortium of 4 bacterial strains: *Sphingobacterium suaeda* T47 *Pseudomonas* sp. DN 13–01 *Bacillus cereus* 263AG5 *Bacillus pumilus* X22	Date palm seedlings (cultivar not specified)	AMF + PGPR co-inoculation vs. single inoculants: ×3 mycorrhizal colonization intensity Strong activation of phenylpropanoid pathway (PAL activity, polyphenols, lignin) Marked reduction in disease incidence, severity, and mortality Synergistic effect (combination > AMF alone or PGPR alone)	Greenhouse conditions AMF + PGPR co-inoculation vs. single inoculants Duration: Not specified	Ziane et al. (2023) [114]
Biotic (Foa)	*G. intraradices* (now *Rhizophagus irregularis*) (isolated from southern Morocco; multiplied on barley as host)	None	Seedlings cv. ‘Jihel’ (highly susceptible)	AMF + *Foa* vs. *Foa*-only control: ~×2 leaf phosphorus concentration Increased potassium uptake Increased peroxidase activity Increased total phenols content Reduced disease incidence 85% successful AMF colonization rate	Greenhouse conditions AMF root inoculation before *Foa* challenge Duration: 4 months post-inoculation	Abohatem et al. (2011) [113]

Abbreviations: AMF, arbuscular mycorrhizal fungi; PGPR, plant growth-promoting rhizobacteria; MDA, malondialdehyde; H_2_O_2_, hydrogen peroxide; SOD, superoxide dismutase; CAT, catalase; APX, ascorbate peroxidase; POD, peroxidase; G-POD, guaiacol peroxidase; PPO, polyphenol oxidase; PAL, phenylalanine ammonia-lyase; GST, glutathione S-transferase; RWC, relative water content; *Foa*, *Fusarium oxysporum* f. sp. *albedinis*; RH, relative humidity; FC, field capacity.

## 5. Molecular Mechanisms of AMF-Mediated Stress Resilience in Date Palm: Insights from Other Plant–Fungal Symbiosis Systems

The physiological benefits of AMF symbiosis, such as enhanced nutrient uptake and improved water relations, are well-documented. These advantages are underpinned by a complex molecular dialog between the host plant and the fungus. Understanding this interaction at the transcriptomic, proteomic, and metabolomic levels is a key frontier in mycorrhizal research, offering the potential to precisely harness and optimize the symbiosis. This section reviews the current body of knowledge on the molecular changes induced by mycorrhizal and other beneficial fungi in date palm, providing critical insights into the genetic and biochemical basis of its enhanced stress resilience.

### 5.1. Transcriptomic Perspectives on AMF–Plant Interactions

Direct transcriptomic, proteomic, and functional genomic studies investigating AMF–date palm interactions are currently scarce. Consequently, the molecular mechanisms discussed in this section are inferred largely from evidence obtained in other systems: other AMF colonizing different plant hosts, and, for comparison, the taxonomically distinct beneficial fungus Serendipita indica, included here not as an AMF analog but for the convergent stress-response pathways it shares with mycorrhizal symbioses. None of this evidence constitutes direct proof of these mechanisms in date palm; rather, it provides a conceptual framework for identifying candidate pathways that warrant future validation. Future date palm-specific omics studies will be required to confirm these hypotheses.

Direct transcriptomic studies of the AMF–date palm interaction remain unavailable, leaving a critical gap between the well-documented physiological benefits and the underlying molecular mechanisms. Current understanding must therefore be inferred from transcriptomic analyses in other AMF–plant systems. In *Asparagus officinalis* L., a perennial monocot, Zhang et al. (2019) [122] identified 6019 novel genes based on reference-guided assembly under salinity stress. Among the responses, 455 differentially expressed genes (DEGs) were specifically regulated by AMF inoculation, primarily linked to internal cell environment adjustments and ROS-scavenging mechanisms, water and nutrient transport, and cell wall modification. Twenty-three differentially expressed transcription factors—including MYB, bHLH, WRKY, and NAC families—are modulated under salinity stress; notably, the AoNAC77 gene is strongly upregulated under both saline conditions and AMF inoculation [123]. Furthermore, arbuscular mycorrhizal fungi play a key role in biotic stress mitigation. When evaluating the biocontrol effect of *F. mosseae* against the soybean root rot pathogen *F. oxysporum*, transcriptomic profiling of the pathogen revealed massive transcriptional reprogramming, with thousands of differentially expressed genes (DEGs) enriched in pathways such as phenylpropanoid biosynthesis, MAPK signaling, and ABC transporters [124]. These cross-species data suggest three coordinated regulatory modules likely operating in date palm: (i) ROS-scavenging genes controlled by stress-responsive transcription factors; (ii) nutrient and water transporters (phosphate, ammonium, aquaporin families); and (iii) phenylpropanoid-mediated cell wall reinforcement. The availability of a high-quality *P. dactylifera* reference genome [125] now opens the way for direct RNA-seq validation of these responses in mycorrhizal date palm.

Direct evidence specifically on AMF in *P. dactylifera* remains absent, but recent work on the related endophytic fungus *Serendipita indica* (Sav. Verma, Aj. Varma, Rexer, G. Kost & P. Franken) M. Weiss, F. Waller, A. Zuccaro & Selosse provides a relevant comparison. Ahmad et al. (2024) [126] conducted a comparative transcriptomic analysis of *S. indica*-colonized date palm roots under high salinity, revealing a massive transcriptional reprogramming with thousands of differentially expressed genes (DEGs). A significant proportion of these transcripts (approximately 60–65%) was strongly upregulated. Functional enrichment analyses revealed the activation of stress perception and signaling pathways, including calcium-dependent protein kinases (CDPKs), mitogen-activated protein kinases (MAPKs), and major transcription factor families such as MYB, WRKY, NAC, and bZIP. Genes involved in osmotic adjustment (e.g., P5CS and proline biosynthesis), ion transport (Na^+^/H^+^ antiporters, HKT transporters, and plasma membrane H^+^-ATPases), and antioxidant defense systems (SOD, CAT, APX) were also significantly induced. These transcriptional changes were associated with improved ionic homeostasis, reduced oxidative stress, and enhanced physiological performance under saline conditions. Although *S. indica* differs from AMF, complementary proteomic studies in date palm have shown differential accumulation of salt stress-related proteins involved in ion transport and signaling [127,128], suggesting that AMF likely operate through similar broad regulatory frameworks including SOS-related pathways [120,129,130].

### 5.2. Proteomic and Metabolomic Evidence

Direct proteomic and metabolomic studies on the AMF–date palm symbiosis are currently lacking. Nevertheless, broader meta-analyses across multiple plant species (*P. dactylifera* was not included) provide a solid foundation for predicting molecular responses in date palm. In a comprehensive meta-analysis, Domingo et al. (2023) [131] identified 4262 plant proteins differentially abundant in AMF-colonized hosts, revealing extensive proteome remodeling both locally in roots and systemically in shoots and leaves.

Key trends emerging from these studies include upregulation of nutrient transporters (e.g., phosphate and ABC transporters) and modulation of core cellular machinery, such as ribosomal proteins and translation factors, indicating enhanced protein synthesis capacity to support symbiosis. In *Poncirus trifoliata* (L.) Raf. colonized by *R. irregularis*, ~80% of 365 differentially expressed proteins were upregulated, involving carbohydrate and fatty acid metabolism, one-carbon metabolism, and other central pathways. Many of these proteins corresponded to transcript-level changes, highlighting coordinated genome-to-proteome responses.

Proteomic shifts translate into measurable physiological benefits under stress. AMF-inoculated plants often show enhanced expression of photosynthetic enzymes and stress-related proteins, correlating with improved chlorophyll content, photosynthetic performance, and antioxidant capacity. Metabolomic analyses further reveal accumulation of compatible solutes (e.g., proline, glycine betaine), sugars, amino acids, and organic acids, supporting osmotic adjustment, membrane integrity, and redox homeostasis.

Projecting these mechanisms onto date palm, AMF symbiosis likely enhances ion homeostasis, stress signaling, antioxidant defenses, and metabolic flexibility, thereby improving resilience under salinity and other abiotic stresses. This projection is supported by transcriptomic studies of beneficial fungi in date palm (e.g., Ahmad et al., 2024) [126], which demonstrated upregulation of Na^+^/K^+^ transporters, SOS1 antiporter, antioxidant enzymes, and stress-responsive transcription factors.

## 6. AMF–Date Palm Interaction: Biotechnological Applications

Research on the AMF–date palm symbiosis has enabled numerous biotechnological applications aimed at improving the sustainability and productivity of date palm cultivation. This section explores two key areas of innovation including the production of high-quality mycorrhizal inoculants using both traditional and monoxenic culture and the mycorrhization of date palm in vitro plants.

### 6.1. Production of High-Quality Mycorrhizal Inoculants

The primary and most direct application of this research is the production of high-quality AMF inoculants for use in commercial nurseries. Studies have consistently shown that AMF-inoculated date palm seedlings exhibit significantly higher survival rates, enhanced growth, and greater robustness, making them better prepared for the harsh conditions of field transplantation [48,50]. Two main strategies are currently employed for inoculum production, each with its own advantages: traditional soil-based pot cultures and advanced in vitro techniques.

#### 6.1.1. Soil-Based Propagation of Indigenous AMF Strains

The use of indigenous AMF represents a key strategy for developing effective inoculants in date palm cultivation. Native strains are typically better adapted to local edaphic and climatic constraints, such as alkaline soils, salinity, and water scarcity, and therefore often perform better than non-adapted commercial inocula. For this reason, several research programs in arid regions, particularly in Morocco and Oman, have implemented a “from isolation to application” approach aimed at developing locally adapted AMF-based biofertilizers [35,47]. This strategy generally involves three stages. First, native AMF are isolated from the rhizosphere of healthy date palms in oasis ecosystems. Second, the isolates are identified and screened for symbiotic efficiency and stress tolerance, allowing the selection of highly effective species. Among the taxa most frequently selected are *F. mosseae* and *R. irregularis*, which are widely reported for their ecological adaptability and strong symbiotic performance.

Selected strains are then mass-produced in soil-based pot cultures using highly mycotrophic host plants such as leek (*Allium porrum*), sorghum (*Sorghum bicolor*), or maize (*Zea mays*). These plants are grown in sterilized substrates, typically sand–soil mixtures, where AMF proliferate over several months and generate a dense network of spores, hyphae, and colonized root fragments. The resulting inoculum generally contains 800–1200 infective propagules per 100 g of substrate. In commercial nurseries, this inoculum is incorporated into the growth substrate, typically at 10–15% (*v*/*v*), during the acclimatization phase of date palm plantlets, particularly those produced through micropropagation. Early colonization ensures the establishment of a stable symbiosis prior to field transplantation, thereby improving nutrient acquisition, plant vigor, and tolerance to environmental stresses.

#### 6.1.2. In Vitro Mass Production Using Hairy Root Cultures

Although soil-based propagation remains widely used, in vitro production systems based on hairy root cultures represent a major technological advancement in AMF biotechnology. This technique was first described by Declerck et al. (1998) [132], who successfully propagated *R. irregularis* on Ri T-DNA transformed carrot roots under sterile conditions. Since then, the method has been refined and extended to other host systems, such as chicory (*Cichorium intybus*), and to additional AMF taxa including *Claroideoglomus* and *Gigaspora* species [133]. Hairy root cultures allow AMF to be maintained in axenic in vitro environments, enabling continuous multiplication of fungal propagules while preventing microbial contamination. Compared with conventional pot cultures, this system offers several advantages [134,135]. First, sterile culture conditions ensure high purity and biosafety, producing inocula free from pathogens or unwanted microorganisms. Second, optimized systems allow high-density production, reaching approximately 15,000–25,000 spores per gram of substrate within 3–5 months, far exceeding densities typically obtained in soil-based cultures. Finally, in vitro systems enable precise control of environmental parameters, including nutrient composition, pH, and temperature, resulting in highly standardized inoculum quality. Although date palm itself is not used to generate hairy root cultures, AMF inoculum produced on model hosts such as carrot or chicory can subsequently be applied to date palm seedlings or in vitro plants. This approach therefore ensures a reliable supply of high-quality, pathogen-free AMF propagules, which is essential for large-scale inoculation programs.

### 6.2. Mycorrhization of Date Palm In Vitro Plants: A Technological Breakthrough

Recent advances have enabled the in vitro mycorrhization of date palm in vitro plants, representing a major step forward in mycorrhizal biotechnology. Conventional micropropagation systems produce genetically uniform and pathogen-free plants, but these plantlets are typically devoid of beneficial microbial symbionts. Introducing AMF during the early stages of plant development therefore offers an opportunity to generate plants that are both elite and symbiotically competent.

El Hilali et al. (2021) [136] reported the first successful in vitro colonization of date palm plantlets by AMF. Using a two-compartment culture system, in vitro plants of the cultivar ‘Boufeggouss’ were co-cultivated with a consortium of native AMF strains. The fungi were initially maintained on transformed carrot roots acting as a donor system, from which hyphae extended to colonize the roots of date palm plantlets within approximately 10 weeks.

Although aboveground growth remained unchanged during the in vitro phase, mycorrhization significantly altered root system architecture. Colonized plantlets exhibited greater total root length and increased formation of secondary and tertiary roots, resulting in a more developed root network. This response likely results from molecular signaling between the symbiotic partners. In particular, fungal signaling molecules known as mycorrhizal lipochitooligosaccharides (Myc-LCOs) have been shown to stimulate root branching and development [137]. Integrating mycorrhization into the micropropagation process offers several advantages. It enables the production of pathogen-free, symbiotically competent plantlets, improves survival and vigor during the critical acclimatization stage, and provides a standardized protocol that could incorporate mycorrhizal status as a quality criterion in certification programs for date palm planting material.

Overall, the integration of AMF into the micropropagation pipeline, particularly during the ex vitro acclimatation and nursery stages, represents a promising strategy for producing elite date palm plants. While in vitro inoculation remains technically challenging due to sterility constraints, introducing AMF into the potting substrate during the early nursery phase aligns perfectly with root system development. This early symbiosis significantly reduces weaning mortality, enhances nutrient uptake, and ensures that micropropagated plantlets are pre-adapted for field establishment and stress tolerance in arid agroecosystems, thereby supporting more sustainable and resilient cultivation systems.

### 6.3. From Production to Commercialization: Formulation Challenges and Opportunities

Translating the production methods described above into efficient, commercially viable AMF-based products requires overcoming several formulation-related challenges beyond propagule production itself. A first challenge concerns carrier selection and shelf-life: AMF propagules must be delivered in a stable, farmer-friendly formulation, such as peat-, vermiculite-, or clay-based carriers, or, increasingly, encapsulated formulations (e.g., alginate beads) that preserve propagule viability during storage and transport under the often high ambient temperatures characteristic of oasis regions. A second, closely related challenge concerns quality control and regulatory standardization: unlike chemical fertilizers, biofertilizers based on AMF currently lack internationally harmonized standards for guaranteeing minimum viable propagule density, purity, and absence of contaminants, which complicates both commercial certification and farmer confidence [135]. A third challenge is the frequently observed gap between the strong efficacy of AMF inoculants under controlled greenhouse conditions and their more variable performance in the field, where indigenous microbial competition, soil heterogeneity, and inconsistent application practices can all reduce establishment success (Section 7.2). Despite these challenges, several opportunities are emerging to accelerate commercial translation. Encapsulation and carrier-optimization technologies can improve propagule shelf-life and enable slow-release or targeted delivery at planting. Combining AMF with compatible PGPR or organic amendments, as reviewed in Section 4.2, can also enhance and stabilize field performance while offering value-added, multifunctional biofertilizer products. Finally, the increasing availability of locally adapted, cultivar-matched AMF consortia (Section 7.2) offers a pathway toward premium, differentiated products tailored to specific oasis agroecosystems, potentially improving both agronomic performance and economic viability for smallholder date palm producers [84,135].

## 7. Conclusions and Future Perspectives

### 7.1. A Powerful Natural Alliance

More than eight decades of research, beginning with the pioneering observations of Sabet in 1940 [32], have demonstrated the fundamental role of AMF in the biology of the date palm. Rather than being passive inhabitants of the rhizosphere, AMF function as active symbiotic partners that enhance the resilience and physiological performance of this emblematic desert species.

Biogeographic studies have also revealed region-specific AMF taxa, particularly in North African and Arabian oasis ecosystems, highlighting the date palm rhizosphere as an important reservoir of microbial genetic diversity with significant biotechnological potential. In addition, the body of research reviewed here demonstrates that AMF symbiosis contributes to three key dimensions of date palm functioning: (i) improved nutrition and growth through enhanced P and micronutrient acquisition; (ii) substantial tolerance to abiotic stresses through ion regulation, osmotic adjustment, and antioxidant defenses, with mycorrhizal plants exhibiting better water relations, higher biomass under drought, and favorable K^+^/Na^+^ ratios under salinity; and (iii) bioprotection against Bayoud disease via priming of systemic defenses including phenolic compound accumulation and increased peroxidase activity, with microbial consortia (AMF + PGPR) further amplifying all three effects. The practical translation of this knowledge has already begun through the development of native AMF inoculants produced using conventional trap cultures or advanced in vitro propagation systems.

### 7.2. Future Perspectives: From Descriptive Studies to Molecular Understanding

Despite the progress made in understanding the ecological and physiological benefits of AMF in date palm cultivation, several key research gaps remain.

A major priority is the development of cultivar-specific inoculation strategies. Future studies should move beyond generalized inoculants and aim to identify the most effective combinations of AMF species and beneficial bacteria for specific date palm cultivars, particularly those of high economic value or threatened by environmental stress and disease.

Another important frontier concerns the molecular mechanisms underlying the AMF–date palm interaction. To date, most studies on this symbiosis remain physiological or agronomic, while integrated multi-omics investigations (transcriptomics, proteomics, and metabolomics) are still extremely limited or absent for the AMF–date palm system. The recent availability of a high-quality reference genome for *P. dactylifera* [125] represents a major opportunity to bridge this gap and transition from descriptive studies toward predictive and functional genomics. Future research should therefore employ large-scale transcriptomic and proteomic approaches to identify the genes and proteins activated during mycorrhizal colonization and during AMF-mediated stress tolerance.

Such datasets could enable genome-wide association studies aimed at identifying molecular markers linked to desirable agronomic traits, including effective mycorrhizal responsiveness, improved nutrient uptake, and efficient activation of defense pathways. These markers could then be incorporated into marker-assisted selection programs, allowing breeders to rapidly screen large numbers of seedlings and select individuals genetically predisposed to form highly efficient mycorrhizal symbioses.

A related and important limitation of the current evidence base concerns experimental conditions: as summarized in Table 2, nearly all of the quantitative benefits reported in this review were obtained under greenhouse or nursery conditions, where environmental variability is minimized and root colonization can be tightly controlled; only one of the studies compiled in Table 2 (Akensous et al., 2022 [120]) was conducted under field conditions. Consequently, the magnitude of AMF-induced benefits observed under controlled conditions may not be fully reproducible in commercial plantations, where soil heterogeneity, native microbial competition, irrigation practices, and climatic variability are far less constrained. Differences among AMF species, inoculum quality, host genotype, and indigenous microbial communities further contribute to variable outcomes across studies. Further research is therefore needed not only to optimize the large-scale production of high-quality AMF inoculum, but also to validate their agronomic performance through well-replicated, multi-site, long-term field trials under real oasis conditions, rather than relying primarily on short-term greenhouse assays. Advances in in vitro culture systems, such as hairy root culture technologies, offer promising solutions for producing pathogen-free and standardized inocula.

Beyond single symbiotic interactions, future work should also adopt a holobiont perspective, considering the date palm together with its entire associated microbiome. Understanding the interactions between AMF, PGPR, and endophytic fungi could facilitate the design of multifunctional microbial consortia capable of simultaneously improving nutrition, stress tolerance, and disease resistance.

Finally, in the context of climate change, it will be crucial to investigate the performance of the AMF–date palm symbiosis under future environmental scenarios, including elevated CO_2_ concentrations, extreme temperatures, and increasing soil salinity. Identifying “climate-smart” fungal symbionts adapted to such conditions will be essential to maintain sustainable date palm production in arid and semi-arid regions.

## 8. Concluding Remarks

Overall, current knowledge clearly demonstrates that AMF represent a powerful natural ally for the date palm, improving plant nutrition, stress tolerance, and disease resistance. However, a deeper mechanistic understanding of this symbiosis, particularly at the transcriptomic, proteomic, and metabolomic levels, remains largely unexplored. Integrating ecological studies with modern genomic and multi-omics approaches will be crucial to fully harness the potential of this ancient symbiosis and to develop innovative, sustainable strategies for safeguarding date palm cultivation and the fragile oasis ecosystems that depend on it.

## Figures and Tables

**Table 1:** Diversity and biogeography of AMF associated with date palm rhizospheres across major date-growing regions, based on studies published between 2006 and 2025. Studies are organized by geographic region and ordered chronologically within each region. Species names follow the current nomenclature according to Schüßler (2025); taxonomic authorities are provided for the first mention of each species.

Geographic Location	AMF Family/Genus	Representative Species (with Taxonomic Authority)	Occurrence and Remarks	Isolation Method	Identification Method	Reference
North Africa						
Ziz Valley, Morocco	Glomeraceae/*Glomus* Glomeraceae/*Funneliformis* Acaulosporaceae/*Acaulospora* Gigasporaceae/*Scutellospora*	*Glomus constrictum* Trappe; *G. aggregatum* N.C. Schenck & G.S. Sm.; *G. macrocarpum* Tul. & C. Tul.; *G. fasciculatum* (Thaxt.) Gerd. & Trappe *Funneliformis mosseae* (T.H. Nicolson & Gerd.) C. Walker & A. Schüßler *Acaulospora* spp. *Scutellospora* spp.	Frequency of occurrence (% of sites where detected, out of 10 sampled sites): *G. constrictum*: 70% (7/10 sites) *Acaulospora* sp.: 50–70% (5–7/10 sites) *F. mosseae*: 50% (5/10 sites) *G. aggregatum*: 50% (5/10 sites) *G. macrocarpum*: 50% (5/10 sites) *G. fasciculatum*: 40% (4/10 sites) *Scutellospora* spp.: 10% (1/10 sites)	Wet sieving Spore extraction	Spore morphotyping	Bouamri et al. (2006) [34]
Tafilalet (Ziz Valley), Morocco	Glomeraceae/*Glomus* Glomeraceae/*Funneliformis* Glomeraceae/*Rhizophagus* Glomeraceae/*Claroideoglomus* Acaulosporaceae/*Acaulospora* Gigasporaceae/*Scutellospora*	*Glomus aggregatum* N.C. Schenck & G.S. Sm.; *G. constrictum* Trappe; *G. fasciculatum* (Thaxt.) Gerd. & Trappe; *G. macrocarpum* Tul. & C. Tul. *Funneliformis mosseae* *Rhizophagus irregularis* (Błaszk., Wubet, Renker & Buscot) C. Walker & A. Schüßler *Claroideoglomus etunicatum* (W.N. Becker & Gerd.) C. Walker & A. Schüßler *Acaulospora* spp. *Scutellospora* spp.	Frequency of occurrence (out of 10 sites): *G. aggregatum*, *G. constrictum*: 8/10 sites *Acaulospora* spp. (Sp.1, sp.2): 8/10 sites *G. fasciculatum*, *G. macrocarpum*, *F. mosseae*: frequent *R. irregularis*, *C. etunicatum*: less frequent *Scutellospora* spp.: rare (1–2/10 sites) *Glomus* genus = 60% of total species detected.	Wet sieving Spore extraction	Spore morphotyping	Bouamri et al. (2014) [51]
Tafilalet & Zagora, Morocco	Glomeraceae/*Glomus* Acaulosporaceae/*Acaulospora* Entrophosporaceae/*Entrophospora*	*Glomus clarum* T.H. Nicolson & N.C. Schenck; *Glomus* sp. *Acaulospora denticulata* Sieverd. & S. Toro *Entrophospora kentinensis* C.G. Wu & Y.S. Liu	Frequency of occurrence (across sampled sites): *G. clarum*: 60% *Glomus* sp.: 20% *A. denticulata*: 1% *E. kentinensis*: 1%	Wet sieving Spore extraction	Spore morphotyping	Sghir et al. (2014) [52]
Drâa-Tafilalet Oasis, Morocco	Glomeraceae/*Pervetustus* Glomeraceae/*Septoglomus* Glomeraceae/*Claroideoglomus* Glomeraceae/*Funneliformis* Glomeraceae/*Albahypha* Glomeraceae/*Rhizophagus*	*Pervetustus simplex* Błaszk., Chwat & Symanczik *Septoglomus xanthium* (Błaszk., Niezgoda & Goto) Błaszk., Niezgoda & Goto *Claroideoglomus etunicatum* *Funneliformis mosseae* *Albahypha drummondii* (T.H. Nicolson & Schenck) Oehl, Stutz & Sieverding *Rhizophagus irregularis*	All six species detected Frequency not specified	Wet sieving Spore extraction	Spore morphotyping Molecular identification (ITS/LSU)	El Hilali et al. (2022) [48]
Figuig Oasis, Morocco	Glomeraceae/*Rhizophagus* Glomeraceae/*Funneliformis* Glomeraceae/*Sclerocystis* Acaulosporaceae/*Acaulospora* Gigasporaceae/*Scutellospora*	*Rhizophagus* sp. *Funneliformis* sp. *Sclerocystis* sp. *Acaulospora* sp. *Scutellospora* sp.	Relative abundance: *Rhizophagus*: 70–90% (dominant) *Acaulospora*/*Scutellospora*: 10–20% *Sclerocystis*: <10% (rare) *Funneliformis*: present	Wet sieving Spore extraction	Spore morphotyping	Gagou et al. (2023) [53]
Figuig Oasis, Morocco	Glomeraceae/*Rhizophagus* Glomeraceae/*Glomus*	*Rhizophagus* spp. *Glomus* spp.	NA (Not applicable)	Spore extraction Single-spore culture	Spore morphotyping Molecular identification (SSU/ITS/LSU)	Gagou et al. (2025) [45]
Djerid palm groves, Tunisia	Glomeraceae/*Funneliformis* Glomeraceae/*Glomus* Glomeraceae/*Rhizophagus* Glomeraceae/*Sclerocystis* Acaulosporaceae/*Acaulospora* Gigasporaceae/*Dentiscutata* Gigasporaceae/*Scutellospora*	*Funneliformis mosseae* *Glomus constrictum* Trappe; *G. tortuosum* N.C. Schenck & G.S. Sm.; *Glomus* sp. *Rhizophagus irregularis* *Sclerocystis rubiformis* (Gerd. & Trappe) R.T. Almeida & N.C. Schenck *Acaulospora cavernata* Błaszk. *Dentiscutata calospora* (T.H. Nicolson & Gerd.) Sieverd., F.A. Souza & Oehl	Marked seasonal variation in spore frequency Occurrence order: spring < summer < winter < autumn	Wet sieving Spore extraction	Spore morphotyping	Zougari-Elwedi et al. (2016) [54]
Tozeur oases, Tunisia	Glomeraceae/*Funneliformis* Glomeraceae/*Rhizophagus* Glomeraceae/*Dominikia* Glomeraceae/*Albahypha*	*Funneliformis coronatus* (Giovann.) C. Walker & A. Schüßler *Rhizophagus irregularis* *Dominikia disticha* (T.H. Nicolson & Schenck) Oehl, Błaszk. & Sieverd. *Albahypha drummondii* (Błaszk. & Renker) Sieverd., Oehl, B.T. Goto & G.A. Silva	Colonization rate negatively correlated with soil salinity	Wet sieving Spore extraction	Spore morphotyping Molecular identification (ITS/LSU)	Chebaane et al. (2020) [49]
Ouargla, Algeria	Glomeraceae/*Glomus* Glomeraceae/*Rhizophagus* Glomeraceae/*Funneliformis* Glomeraceae/*Claroideoglomus* Gigasporaceae/*Scutellospora* Gigasporaceae/*Gigaspora* Gigasporaceae/*Racocetra* Acaulosporaceae/*Acaulospora*	*Glomus* spp., *Rhizophagus* spp., *Funneliformis* spp., *Claroideoglomus* spp., *Scutellospora* spp., *Gigaspora* spp., *Racocetra* spp., *Acaulospora* spp. (species-level identification not reported)	14 AMF morphotypes recorded across 4 sites (frequency by species not specified) *Glomus* and *Scutellospora* reported as dominant genera Spore density up to 345 spores/100 g soil (highest at Kser site) No significant inter-site difference in soil physicochemical properties except calcite content	Spore extraction	Spore morphotyping	Khirani et al. (2020) [55]
Arabian Peninsula						
Southern Oman (Sultanate of Oman)	Glomeraceae/*Glomus* Gigasporaceae/*Dentiscutata* Gigasporaceae/*Racocetra* Acaulosporaceae/*Acaulospora* Paraglomeraceae/*Paraglomus* Ambisporaceae/*Ambispora*	*Glomus sinuosum* (Gerd. & B.K. Bakshi) R.T. Almeida & N.C. Schenck; *G. aggregatum* N.C. Schenck & G.S. Sm.; *G. microaggregatum* Koske, Gemma & P.D. Olexia; *G. eburneum* L.J. Kenn., J.C. Stutz & J.B. Morton; *G. microcarpum* Tul. & C. Tul.; *G. macrocarpum* Tul. & C. Tul.; *C. etunicatum*.; *G. constrictum* Trappe; *Glomus* spp. (Morphotypes OMA2–OMA13) *Dentiscutata calospora* (T.H. Nicolson & Gerd.) Sieverd., F.A. Souza & Oehl *Racocetra gregaria* (N.C. Schenck & T.H. Nicolson) Oehl, F.A. Souza & Sieverd.; *R. fulgida* (Koske & C. Walker) Oehl, F.A. Souza & Sieverd. *Acaulospora spinosa* C. Walker & Trappe *Paraglomus occultum* (C. Walker) J.B. Morton & D. Redecker *Ambispora gerdemannii* (S.L. Rose, B.A. Daniels & Trappe) C. Walker, Vestberg & A. Schüßler	Relative abundance by genus: *Glomus* (dominant): 72% Gigasporaceae (*Dentiscutata*, *Racocetra*): 8% *Acaulospora*: 4% *Ambispora*: 4% *Paraglomus*: 4% Molecular phylotypes (~16%) distantly related to described species (potentially undescribed lineages)	Wet sieving Spore extraction	Spore morphotyping Molecular identification (ITS/LSU/SSU)	Al-Yahya’ei et al. (2011) [35]
Northern Oman (Sultanate of Oman)	Diversisporaceae/*Diversispora* Glomeraceae/*Funneliformis* Glomeraceae/*Claroideoglomus*	*Diversispora spurca* (C.M. Pfeiff., C. Walker & Bloss) C. Walker & A. Schüßler *Diversispora aurantia* Błaszk., Ryszka, Kovács & Buscot *Funneliformis africanum* Oehl, Tchabi, Hountondji, Lawouin, Coyne & Sieverd. *Claroideoglomus drummondii* (Błaszk. & Tadych) C. Walker & A. Schüßler	Occurrence frequency across sampled sites: *D. spurca*: 3 sites (most widespread) *D. aurantia*: 2 sites *F. africanum*: 2 sites *C. drummondii*: 1 site	Wet sieving Single-spore culture	Spore morphotyping Molecular identification (LSU)	Symanczik et al. (2014) [33]
Al-Jamil Farm, Qassim, Saudi Arabia	Glomeraceae/*Glomus*	*Glomus* spp. (species-level identification not reported)	Root colonization parameters (range across sampled plants): Total colonization rate (TC): 28–72% Vesicular colonization (VC): 31–60% Arbuscular colonization (AC): 26–59% Spore density: 95–130 spores per 100 g dry soil	Wet sieving Spore extraction	Spore morphotyping	Khaliel & Abou-Heilah (1985) [56]
Qatar (arid-land sites)	Glomeraceae/*Claroideoglomus* Glomeraceae/*Rhizophagus* Diversisporaceae/*Diversispora*	*Claroideoglomus drummondii* *Rhizophagus irregularis* *C. claroideum* (N.C. Schenck & G.S. Sm.) C. Walker & A. Schüßler *Diversispora aurantia* Note: *Phoenix dactylifera* not directly sampled; data provide regional context for the Persian Gulf	Most widespread taxa: *C. drummondii* and *R. irregularis* Least frequent taxa: *C. claroideum* and *D. aurantia*	Wet sieving Spore extraction	Molecular identification (ITS)	Alrajhei et al. (2022) [36]

Abbreviations: SSU, small subunit ribosomal DNA; ITS, internal transcribed spacer; LSU, large subunit ribosomal DNA; PCR, polymerase chain reaction.

## Data Availability

The original contributions presented in this study are included in the article. Further inquiries can be directed to the corresponding author.
